# Drusen in AMD from the Perspective of Cholesterol Metabolism and Hypoxic Response

**DOI:** 10.3390/jcm13092608

**Published:** 2024-04-29

**Authors:** Norimitsu Ban, Ari Shinojima, Kazuno Negishi, Toshihide Kurihara

**Affiliations:** 1 Laboratory of Aging and Retinal Biology, Keio University School of Medicine, Tokyo 160-8582, Japan; nban@keio.jp; 2 Department of Ophthalmology, Keio University School of Medicine, Tokyo 160-8582, Japan; ari.shinojima@keio.jp (A.S.); kazunonegishi@keio.jp (K.N.); 3 Laboratory of Photobiology, Keio University School of Medicine, Tokyo 160-8582, Japan

**Keywords:** drusen, age-related macular degeneration (AMD), cholesterol metabolism, hypoxic response, vascular endothelial growth factor (VEGF), hypoxia inducible factor (HIF)

## Abstract

Drusen are one of the most characteristic pathologies of precursor lesion of age-related macular degeneration (AMD). Drusen comprise a yellowish white substance that accumulates typically under the retinal pigment epithelium (RPE), and their constituents are lipids, complement, amyloid, crystallin, and others. In the past, many researchers have focused on drusen and tried to elucidate the pathophysiology of AMD because they believed that disease progression from early AMD to advanced AMD might be based on drusen or drusen might cause AMD. In fact, it is well established that drusen are the hallmark of precursor lesion of AMD and a major risk factor for AMD progression mainly based on their size and number. However, the existence of advanced AMD without drusen has long been recognized. For example, polypoidal choroidal vasculopathy (PCV), which comprises the majority of AMD cases in Asians, often lacks drusen. Thus, there is the possibility that drusen might be no more than a biomarker of AMD and not a cause of AMD. Now is the time to reconsider the relationship between AMD and drusen. In this review, we focus on early AMD pathogenesis based on basic research from the perspective of cholesterol metabolism and hypoxic response in the retina, and we discuss the role of drusen.

## 1. Age-Related Macular Degeneration (AMD) and Drusen: A Cause or a Biomarker?

Age-related macular degeneration (AMD) is the leading cause of blindness in developed countries. Clinically, it is classified as early or intermediate stage to advanced stage. Early stage AMD includes clinical signs such as drusen and abnormalities of the retinal pigment epithelium [1]. Advanced-stage AMD can be neovascular (or wet) type characterized by macular neovascularization (MNV) [2] or atrophic (or dry) type characterized by geographic atrophy (GA) lesions in the macula, which accompanies the degeneration of photoreceptor cells [1].

In the conventional classification of early or intermediate AMD, early AMD is defined by the presence of medium drusen >63 μm and ≤125 μm and no pigment abnormalities [3], and intermediate AMD is defined by large drusen >125 μm or any pigment abnormalities [3]. 

Advanced AMD is defined by the presence of signs indicating either neovascular or atrophic AMD. In the classification of neovascular AMD, subtypes of MNV are classified according to the site of suspected invasion into the retina. Type 1 MNV arises when choroidal neovascularization proliferation occurs below the retinal pigment epithelium, and it corresponds to occult choroidal neovascularization with a poorly defined pattern of leakage on fluorescein angiography. Type 2 MNV refers to choroidal neovascularization proliferation above the retinal pigment epithelium in the subretinal space, and it corresponds to classic choroidal neovascularization with intense fluorescein leakage. Type 3 MNV occurs when the retinal circulation is involved, with an anastomosis between the choroidal and retinal circulations [4]. 

In the conventional classification of neovascular AMD, there are two subtypes in addition to typical neovascular AMD. One is polypoidal choroidal vasculopathy (PCV) [5], which is more common in Asians [6]. The other is retinal angiomatous proliferation (RAP) [4] or type 3 MNV [2]. Surprisingly, PCV can account for 50% of wet AMD cases in Asians but only 8–13% in white cases [6]. When we focus on drusen, it is well known that PCV often lacks drusen. In addition, the new disease concept of macular neovascularization (MNV) without drusen that includes pachychoroid neovasculopathy has been proposed [7], although we do not discuss the new classification, such as pachychoroid neovasculopathy, in this review because the disease concept and definition are still being discussed.

**Figure 1:**
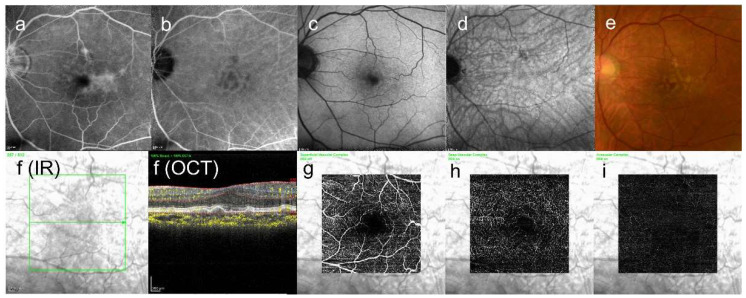
(**a**) FA image acquired at 6 min, diffuse fluorescence can be seen; (**b**) ICGA image, abnormal hypofluorescence contrast became clearer acquired at 13 min; (**c**) short-wavelength fundus autofluorescence image, it is hard to see abnormal fluorescence; (**d**) near-infrared autofluorescence image, abnormal hypofluorescence can be seen at the upper side above the fovea; (**e**) color fundus photograph, drusen can be seen at the macular area; (**f**) infrared + OCT angiography (horizontal section) through the drusen; (**g**) superficial vascular segment OCT angiography; (**h**) deep vascular segment OCT angiography; (**i**) avascular segment OCT angiography, (**g**–**i**) where there is no macular neovascularization.

**Figure 2:**
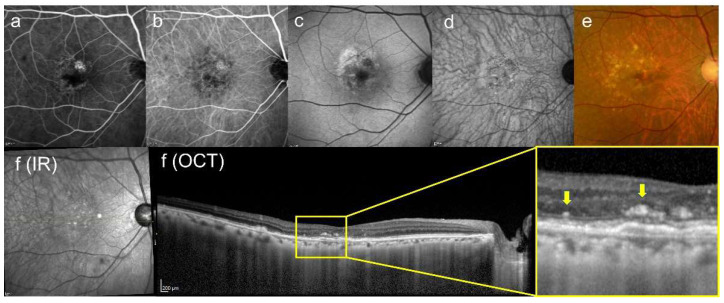
This is a figure of atypical drusen, which is not located under the RPE. (**a**) FA image acquired at 1 min, apparent fluorescence leakage was not seen, but window defect findings were seen; (**b**) ICGA image acquired at 8 min, similar to the findings of (**a**), hypofluorescent lesions were confirmed corresponding to drusen or drusenoid pigment epithelial detachment (PED); (**c**) short-wavelength fundus autofluorescence image, abnormal hyperfluorescence and patchy hypofluorescence can be seen; (**d**) near-infrared autofluorescence image, abnormal fluorescence is rather difficult to detect; (**e**) color fundus photograph, drusen can be seen broadly; (**f**) infrared + OCT angiography (horizontal section) through the drusen, a left yellow arrow indicates untypical pseudo drusen and a right yellow arrow indicates a drusen ooze deposits with a shallow drusenoid PED, which can be seen on above the external limiting membrane.

Drusen are various lipid- and protein-rich extracellular deposits that accumulate typically under the retinal pigment epithelium (RPE) (typical drusen with multimodal imaging are shown in Figure 1). However, recent advances of imaging techniques, such as optical coherence tomography (OCT), revealed that there are some types of atypical drusen that accumulate other than under the RPE (atypical drusen are shown in Figure 2). Particularly, subretinal drusenoid deposits (SDD) accumulate under the neurosensory retina, not under the RPE [8]. Drusen composition has revealed proteins other than RPE remnants (lipofuscin, an autofluorescent pigment formed through the oxidation of unsaturated fatty acids) such as lipids, components of the complement cascade, and its inhibitors, amyloid, immunoglobulins, class II antigens, acute-phase proteins, apolipoproteins (apo) B and E, and others [9,10,11]. In general, soft, large drusen are associated with an increased risk of disease progression mainly based on their size and number [12,13]. At least 40% of drusen’s components are lipids and proteins, the majority of which are esterified cholesterol (EC) and phosphatidylcholine (PC) [14]. The following Table 1 summarizes drusen’s components from previous reports [14,15]. It is reported that copper levels are not distinguishable when detecting drusen components [14]. However, we put copper in Table 1 because the oligomerization of complement factor H is induced by copper, but not calcium or iron, in vitro [16].

There is no doubt that drusen are the most characteristic hallmark of precursor lesion of AMD [1]. One of the reasons is that both neovascular and atrophic AMD can arise from the same precursor lesion, which is characterized by drusen [1], although we are still unclear about the factors that lead to each disease type, neovascular or atrophic. Therefore, in order to establish therapeutics to prevent the progression from early or intermediate AMD to advanced neovascular or atrophic AMD, it is essential to understand the detailed pathophysiology of early AMD and reasonable to focus on drusen, which is the hallmark of precursor lesion of AMD.

In fact, numerous basic research originating from drusen has provided many insights into the pathophysiology of AMD. For example, amyloidβ is one of the componants of drusen that increases VEGF in the RPE, and an accumulation of amyloidβ reproduces features characteristic of human AMD, such as RPE atrophy and basal deposit formation [17]. Other studies also have been focused on the complement, which is another major componant of drusen [18], and the first treatment that recently has been approved by the US Food and Drug Administration for geographic atrophy is the complement inhibitor [19]. As evidenced by these facts, there is a possibility that disease progression from early or intermediate AMD to advanced AMD might be based on drusen, or drusen itself might be upstream of causing AMD.

However, as described above, the existence of advanced AMD without drusen has long been recognized. For example, PCV, which comprises the majority of AMD cases in Asians, often lacks drusen [5,6]. In addition, the incidence and type of drusen in AMD patients also differs between Asians and Caucasians [20]. Thus, there is another hypothesis that drusen might not be a cause of AMD and is no more than a biomarker of AMD.

In this review, we discuss drusen in AMD from the perspective of cholesterol metabolism and hypoxic response based on the animal model of AMD because most clinical studies are descriptive, and it is difficult to judge causal relationships. Compared to clinical studies, the animal model of AMD, especially the gene-edited animal models, is very useful in identifying upstream and downstream factors and cause and result. 

## 2. Risk Factors of AMD and Drusen

In terms of risk factors of AMD, first of all, aging itself is the strongest risk factor. In fact, AMD usually develops in adults older than 60 years of age [21]. For other risk factors, previous studies have shown that smoking, uncontrolled hypertension, and obesity increase the risk of developing AMD [22,23,24]. The aging and risk factor genes of AMD, such as ARMS2, are also reported as risk factors of drusen formation [25]. In general, soft, large drusen are associated with an increased risk of disease progression mainly based on their size and number [12,13], and thus, we can regard the existence of drusen as one of the risk factors of AMD. 

It is clear that AMD is not a monogenic hereditary disease. However, it is considered to have genetic factors because of the differences in prevalence rate and characteristics among the genetic backgrounds [26]. First, AMD is more common in Caucasian people compared to Asian, Hispanic, or Black populations [1]. Second, as described above, typical AMD is the primary type in Caucasians, while PCV is more prevalent in Asians [6].

Recent advances in genome-wide association study (GWAS) technology have revealed some risk factor genes. CFH (complement factor H), ARMS2, and HTRA1 are well-known genes that are strongly associated with the development and progression of AMD [27,28]. Minor allele frequencies, such as CFH and HTRA1, vary among races, which may be the main cause of ethnic differences in AMD expression [26]. Particularly, a common variant in CFH increased by 7.4-fold the risk of developing AMD in individuals homozygous for this allele. It is of crucial significance that CFH is one of the risk factor genes, and drusen consist of components of the complement [29,30,31,32], which supports the hypothesis that drusen’s components, such as complement and components of the complement cascade, including the membrane-attack complex, can cause AMD. Based on this hypothesis, numerous studies and clinical trials on the complement cascade in AMD have been performed [18], and finally, the complement inhibitor has been approved as the first treatment for geographic atrophy, as described above [19]. Actually, complement factors 3 and 5 (C3 and C5) inhibition compared to sham favorably reduce change in square root GA [33].

In addition to CFH, ARMS2, and HTRA1, other genes involved in lipid metabolism, especially the high-density lipoprotein cholesterol (HDL-C) pathway, ABCA1, LIPC, CETP, and LPL, have been found to be involved in the development and progression of AMD [34,35]. It has been reported that adrenomedullin and C3b share a binding site for CFH, which has a dual effect on both molecules and is likely to cause either neovascular AMD or choroidal disease [26]. Based on the fact that drusen consist of lipids, such as oxidized cholesterol, in addition to these variants of cholesterol pathway-associated genes, our research has been focused on the hypothesis that disrupted cholesterol metabolism in the retinal tissue causes AMD.

## 3. Cholesterol Metabolism in AMD

### 3.1. Serum or Plasma Cholesterol in AMD

When considering the pathophysiology of drusen, they resemble the formation of atherosclerotic plaques seen in cardiovascular diseases [30,36,37]. It is also well known that lowering blood cholesterol has an improving effect on atherosclerosis.

Thus, numerous previous studies were undertaken to evaluate the relationship between serum or plasma cholesterol and AMD or the treatment effect of cholesterol-lowering drugs, such as statins. However, many previous studies did not find a significant relationship between serum or plasma lipids and AMD, although several studies of larger size found a significant effect of high HDL on developing AMD [38,39]. In addition, it has been recently reported that ApoB/non-HDL-C is an independent risk factor for typical AMD, and apoB is an independent risk factor for PCV [40]. Low-density lipoprotein cholesterol is significantly higher in patients with PCV when compared with typical AMD [40]. Therefore, consideration should be given to the systemic diseases of AMD patients, and perhaps the results of medical examinations might be consulted.

However, the treatment effect of cholesterol-lowering drugs such as statins is also unclear. Although a small sample study showed that high-dose atorvastatin resulted in the regression of drusen deposits associated with vision [41], and the meta-analysis also showed the protective effect of statin use on both early and advanced AMD [42], these studies are descriptive, and it remains to be elucidated on how circulating cholesterol contributes to drusen formation or AMD pathogenesis.

### 3.2. Tissue Cholesterol Metabolism in the Retina

As described above, the possibility that systemic cholesterol metabolism affects AMD may still be controversial [40]. However, it has become clear that the cholesterol metabolism in the retinal tissues is more important in the pathophysiology of AMD [37,43].

#### 3.2.1. Macrophage/Microglia

Numerous studies have shown that the cellular and humoral components of the innate immune system, including macrophages, contribute to both the development and severity of AMD [43,44,45]. In addition to antigen-presenting and phagocytic rolls, macrophages are also important in cholesterol regulation because they remove cholesterol from peripheral tissues and transport it back to the liver through the bloodstream. This process is termed cholesterol efflux or reverse cholesterol [43]. In this reverse cholesterol transport system, ATP-binding cassette protein A1 (ABCA1) and ATP-binding cassette protein G1 (ABCG1) play crucial roles by effluxing cholesterol from macrophages to extracellular carriers, such as ApoA1 and HDL, respectively. Sene et al. reported that macrophage polarization is also linked to aging, and Abca1 deletion in the macrophage mimics aging and promotes AMD phenotype [43].

**Figure 3:**
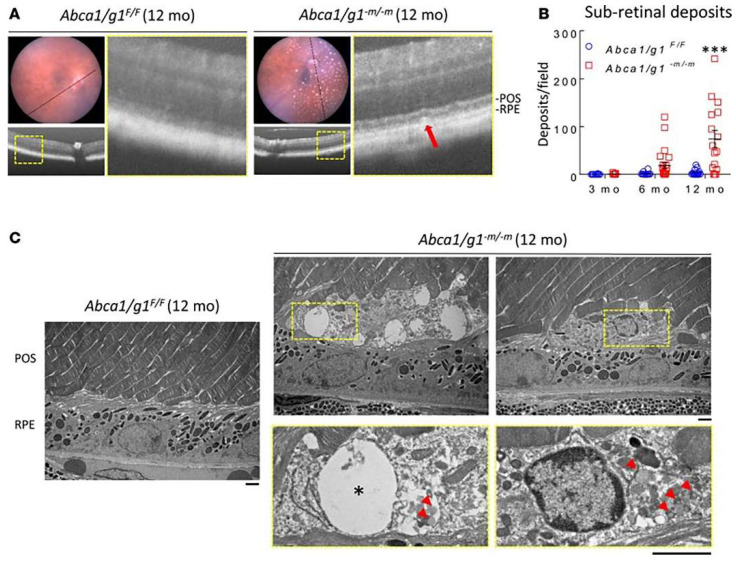
Subretinal deposits contain lipid globules and degenerative vacuoles. (**A**) Representative optical coherence tomography (OCT) images of 12-month-old Abca1/g1F/F and Abca1/g1–m/–m mice retinas. Note the subretinal hyperreflective deposits in Abca1/g1–m/–m mice retinas (red arrow) corresponding to fundus yellowish white dots. POS, photoreceptor outer segment. (**B**) Quantification of subretinal deposits in 3-, 6-, and 12-month-old Abca1/g1F/F and Abca1/g1–m/–m mice retinas. Abca1/g1F/F, *n* = 10, 10, and 15; Abca1/g1–m/–m, *n* = 13, 24, and 16, respectively. *** *p* < 0.001 by 2-way ANOVA with post hoc Bonferroni’s multiple comparison test. (**C**) Representative electron microscopy images of 12-month-old Abca1/g1F/F and Abca1/g1–m/–m mice retinas. Note the vacuoles (asterisk) and lipid globules (red arrowheads) within the subretinal deposit in Abca1/g1–m/–m mice retinas. Scale bar: 1 μm. Values are mean ± SEM.

We generated conditional knock-out mice, wherein both Abca1 and Abcg1 were deleted in macrophages [46]. In this model, there was an age-dependent manifestation of several anatomic and functional AMD disease phenotypes, including subretinal deposits, increased Bruch’s membrane thickening, and accumulation of free cholesterol, cholesterol metabolites, and cholesteryl esters. In addition, this model also showed impaired dark adaptation and rod photoreceptor dysfunction [46] (Figure 3). Other macrophage depletion experiments suggest that blood-derived macrophages contribute to anti-VEGF resistance [47].

These results indicate that cholesterol clearance by macrophages plays a critical role in the early stages of AMD pathogenesis, although whether macrophage or resident microglia play a more crucial role needs to be elucidated.

#### 3.2.2. Photoreceptors (Cones and Rods)

We also generated conditional knock-out mice, wherein both Abca1 and Abcg1 were deleted in the photoreceptors [48]. In this model, knock-out in rod photoreceptors resulted in the age-dependent accumulation of cholesterol metabolites in the outer retina and retinal neurodegeneration but not in the cone photoreceptor-specific knock-out model.

In addition, this model had lipid droplets accumulated in the RPE but not in the neurosensory retina, which was consistent with the fact that one of the major functions of the RPE is phagocytosis of lipid-rich photoreceptor outer segments.

It is also reported that Biallelic variants in CDHR1, a specialized protocadherin highly expressed in cone and rod photoreceptors, result in blindness from shortened photoreceptor outer segments and progressive photoreceptor cell death [49]. This study showed that hypomorphic CDHR1 variants may mimic advanced dry age-related macular degeneration [49]. Thus, it seems that the subsets expressed in cone and rod influence RPE and influence each other.

#### 3.2.3. Retinal Pigmented Epithelium (RPE)

Storti et al. studied the contribution of cholesterol efflux in the RPE by generating a mouse model lacking Abca1 and Abcg1, and prominent intracellular accumulation of lipid droplets was observed in RPE lacking Abca1/Abcg1. Eventually, the conditional knock-out mouse showed reduced RPE and retinal function, retinal inflammation, and RPE/photoreceptor degeneration [50,51], concluding that lipid concentration eventually may become too high in the absence of a functional efflux pathway and may lead to cell death. These phenotypes are also age-dependent, but it seems that the phenotype develops earlier than other macrophage-specific [46] or photoreceptor-specific knock-out models [48]. This was consistent with the fact that the epicenter of AMD is RPE, pathologically and physiologically.

Another study showed that RPE microsomal triglyceride transfer protein (MTP), encoded by the MTTP gene, is critical for apoB-containing lipoprotein synthesis and assembly but is not directly involved in plasma lipoprotein metabolism, suggesting that RPE-specific MTP expression is necessary to establish and maintain retinal lipid homeostasis and visual function [52]. 

These studies showed that the disruption of lipid metabolism in the retinal tissue results in AMD-like phonotypes (drusen-like lesion and retinal degeneration) with age, which seems to support the hypothesis that drusen cause AMD. 

## 4. Hypoxic Response and AMD

To this point, we focused on drusen and disrupted cholesterol metabolism in the retina, and it might support the hypothesis that drusen cause AMD. However, if we focus on advanced AMD, especially neovascular AMD, it is clear that ischemic change and hypoxic response play central roles, which is proven by the clinical efficacy of anti-VEGF antibody treatment [1]. Our research has also been focused on the hypothesis that the hypoxic response causes AMD.

First of all, most living beings on earth use oxygen to conduct their life activities. Although animals have systems that supply oxygen systemically, the mechanism that protects cells and individuals from the disruption of these systems, i.e., anemia and ischemia, is the hypoxic response. When cells are hypoxic, they use the hypoxic response to increase the number of red blood cells, i.e., hematopoiesis, and the number of blood vessels, i.e., angiogenesis [53]. Semenza et al. named the molecule required for cells to increase the hematopoietic factor erythropoietin under hypoxic conditions hypoxia-inducible factor 1 (HIF-1) [54]. Subsequently, HIF was found to be a master regulator of the hypoxic response, controlling not only erythropoietin but also hundreds of genes involved in angiogenesis and metabolic transformation, such as vascular endothelial growth factor (VEGF) [53]. Based on the fact that anti-VEGF injection is the gold standard of pharmacotherapy that has been confirmed to be effective for neovascular AMD, it is clear that the HIF-VEGF pathway is of extreme importance in AMD pathophysiology.

We discovered several HIF inhibitors through extensive screening of food ingredients and found that diets containing these ingredients were effective in reducing the risk of laser-induced choroidal neovascularization in mice [55,56,57,58], and hypoxic response may well be involved in the background of AMD pathogenesis [59]. Then, why does hypoxia occur? In the older eye, lipid-rich particles are present at the choriocapillaris side of the RPE basal lamina. Such a confluent lipid wall of the inner Bruch’s membrane from accumulating lipid particles may eventually lead to isolating the retina from its blood supply [60].

In clinical ophthalmology, abnormal hypofluorescent areas are detected in the late stage of indocyanine green angiography in AMD [61,62] and central serous chorioretinopathy (CSC), which is thought to be lipid deposition from the Bruch’s membrane to the level of the choriocapillaris [63]. In the area with a confluent lipid wall of the inner Bruch’s membrane from accumulating lipid particles, hypoxia may occur because of interference of the supply of oxygen and nutrients from the choroidal side to the retinal side. As a result, hypoxia-inducible factors, such as HIF-1α, may emerge and produce cytokines, such as VEGF, to cause MNV.

In addition, the products of lipid peroxidation can accumulate lipofuscin, a heterogeneous protein-lipid-carbohydrate [64], which can reduce the activity of RPE autophagy [65]. It has also been speculated that a complex combination of pathological excess of reactive oxygen species and oxidative stress and dysfunction of the autophagy pathway in aged RPE cells cause MNV [66]. It is also reported that fibronectin is associated with the formation of MNV. A recent study showed that the expressions of fibronectin and integrin α5β1 were distinctly increased in a laser-induced MNV mouse model and in an RF/6A cytochemically induced hypoxia model [67]. 

Based on the previous literature, it may be reasonable to consider hypoxic response as the most downstream factor in AMD. However, of interest, our conditional knock-out mice targeting the Von Hippel Lindau (VHL)-HIF-VEGF pathway showed not only retinal degeneration but also drusen-like deposits [68,69]. These facts support the hypothesis that hypoxia and hypoxic response in the retina are the main pathophysiologies of advanced AMD, and drusen might be no more than a biomarker of AMD, not the cause of AMD. 

## 5. Discussion and Limitations

Although the presence of drusen was partially the definition of early or intermediate AMD in the conventional classification [3], there was a big controversy because the existence of advanced AMD without drusen, such as PCV, has long been recognized. In addition, the percentage of PCV patients was too high, especially in Asians [70], to be treated as an exception of advanced AMD.

In this review, we described the pathogenesis of drusen and AMD in detail. We especially discussed the possibility that drusen might be no more than a biomarker of AMD, not the cause of AMD.

First, we focused on cholesterol metabolism in the retina based on the fact that drusen consist of lipids such as oxidized cholesterol in addition to these variants of cholesterol pathway-associated genes. Targeting the deletion of cholesterol efflux genes, such as Abca1 and Abcg1, promoted the formation of drusen-like lesions and finally resulted in retinal degeneration with age [46,48,50], which seems to support the hypothesis that drusen cause AMD. However, it can be argued that the retinal degeneration could be caused by an independent pathway other than drusen-like deposit formation.

Second, we focused on hypoxic response in the retina because ischemia of the retina and its hypoxic response are considered to be the most important factors in neovascular AMD. Unexpectedly, however, the conditional knock-out mice targeting the VHL-HIF-VEGF pathway showed not only retinal degeneration but also drusen-like deposits [68,69]. If hypoxic response is the most downstream factor in neovascular AMD, drusen is not necessarily a requirement to develop advanced AMD because hypoxic response itself can promote drusen formation. 

Accordingly, the discussion on whether drusen cause AMD or are no more than a biomarker of AMD remains to be elucidated. However, it is important to note that, even if drusen might be no more than a biomarker of AMD or not the cause of AMD, this does not necessarily mean that drusen are not involved in the pathogenesis of AMD. 

It is reported that parapapillary drusen and macular drusen may not vary significantly in choriocapillaris thickness and density, while RPE cell density and Bruch’s membrane thickness may be higher in parapapillary drusen [71]. Therefore, although it is generally called drusen, the symptoms that occur may differ depending on the location. 

A recent experiment also showed surgical debridement of the RPE results in GA, choroidal neovascularization, and pachychoroid and reproduced all forms of advanced macular degeneration in female domestic pigs [72]. RPE is closely related to the choriocapillaris and photoreceptor cells, and it seems that the effects of loss of the RPE are immeasurable.

As discussed in this review, drusen might be a cause of AMD but might be no more than a biomarker of AMD. Based on these controversial facts, there may be limits to the conclusion of this discussion based solely on the presence or absence of drusen, and it can be misleading to discuss AMD as a whole because each subtype might have a different pathophysiology. Thus, the new disease concept of macular neovascularization (MNV) without drusen that includes pachychoroid neovasculopathy [7] and the new classification based on the presence or absence of pachychoroid and drusen [73] have been proposed, and further investigation will be necessary in the future to fully understand AMD pathophysiology. 

Limitations of this review include a potential bias in the selection of papers, although we selected the articles in not only our publications but also in a wide range of previous papers related to the topic, especially those published in the last three years. Another limitation is that animal models of AMD are not exactly the same as human AMD models. For example, most animal models in this review are mouse models, but a mouse retina does not have macula, and the distribution of rods and cones is different from human retinas. 

## 6. Conclusions

There is no doubt that drusen are one of the most important pathologies of precursor lesions of AMD and might be a key factor in the development of advanced AMD. However, since the existence of advanced AMD without drusen, such as PCV, has long been recognized, there is the possibility that drusen might be no more than a biomarker of AMD, not the cause of AMD. 

Previous basic research has shown that disrupted drusen-related materials, such as complement or cholesterol, cause AMD phenotype including drusen-like deposits. In this sense, drusen might be a cause of AMD. On the other hand, the fact that knock-out mice target the VHL-HIF-VEGF pathway, which is considered to be the most downstream factor in neovascular AMD, also showed drusen-like deposits might indicate that drusen are just a biomarker of AMD. Accordingly, the discussion on whether drusen cause AMD or are no more than biomarkers of AMD remains to be elucidated. 

These developments have been supported by both recent advances in genetically modified animals and in clinical imaging technology, such as OCT and OCT angiography, in addition to the conventional clinical findings, such as fundus examination and fluorescence fundus angiography. In addition, the new concept of disease, including pachychoroid neovasculopathy, and the new classification based on the presence or absence of pachychoroid and drusen will arise further discussion on what is the role of drusen in AMD pathophysiology. It is hoped that the pathophysiology of AMD will be elucidated and a new treatment will be developed based on the fusion of clinical features and basic sciences.

## Figures and Tables

**Table 1:** Components of drusen in human.

Lipids	Neutral lipid	Esterified cholesterol
		Triglyceride
		Fatty acids
		Unesterified cholesterol
	Polar lipid	Sphingomyelin
		Phosphatidylcholine
Proteins	Albumin, serum	
	Amyloid P component, serum	
	Apolipoprotein B, E	
	ATP synthase, H+ transporting, mitochondrial F1 complex, b polypeptide	
	Clusterin	
	Complement component 5	
	Complement component 6	
	Complement component 8, a polypeptide	
	Complement component 8, b polypeptide	
	Complement component 8, c polypeptide	
	Complement component 9	
	Complement factor H	
	Enolase 2 (c, neuronal)	
	Forkhead-associated (FHA) phosphopeptide binding domain 1	
	Major histocompatibility complex, class II, DR a	
	Retinol dehydrogenase 5 (11-cis/9-cis)	
	Scavenger receptor class B, member 2	
	Serum amyloid A1	
	TIMP metallopeptidase inhibitor 3	
	Vitronectin	
Minerals	Zinc	
	Calcium	
	Iron	
	Copper (probably)	

## Data Availability

The data presented in this study are available on request from the corresponding author. The data are not publicly available due to patients’ privacy.
